# Atherosclerotic Abdominal Aortic Aneurysms on Computed Tomography Angiography: A Narrative Review on Spectrum of Findings, Structured Reporting, Treatment, Secondary Complications and Differential Diagnosis

**DOI:** 10.3390/diagnostics15060706

**Published:** 2025-03-12

**Authors:** Roberta Scicolone, Kosmas I. Paraskevas, Giovanni Argiolas, Antonella Balestrieri, Paolo Siotto, Jasjit S. Suri, Michele Porcu, Cesare Mantini, Massimo Caulo, Salvatore Masala, Filippo Cademartiri, Roberto Sanfilippo, Luca Saba

**Affiliations:** 1 Department of Radiology, University of Cagliari, Cagliari, Italy; 2 Department of Vascular Surgery, Red Cross Hospital, Athens, Greece; 3 Department of Radiology, Azienda Ospedaliera Brotzu, Cagliari, Italy; 4 Stroke Division and Monitoring Division, AtheroPointTM, Roseville, CA, USA; 5 Department of CE, Graphic Era Deemed to be University, Dehradun, India; 6 University Centre for Research & Development, Chandigarh University, Mohali, India; 7 Symbiosis Institute of Technology, Nagpur Campus, Symbiosis International (Deemed University), Pune, India; 8 Department of Radiology, “G. D’Annunzio” University, Chieti, Italy; 9 Department of Radiology, University of Sassari, Sassari, Italy; 10 Department of Imaging, IRCCS Synlab SDN, Napoli, Italy; 11 Department of Vascular Surgery, University of Cagliari, Cagliari, Italy

**Keywords:** AAA, CTA, rupture, impending rupture, EVAR, endoleak, inflammatory aneurysm, mycotic aneurysm, AI

## Abstract

Atherosclerotic abdominal aortic aneurysms (AAAs) are a common vascular pathology with significant morbidity and mortality risks. Timely diagnosis, accurate characterization, and standardized reporting are critical for effective management and monitoring of atherosclerotic AAAs. Imaging modalities, particularly computed tomography angiography (CTA), play a pivotal role in the detection, treatment planning, and identification of both primary and secondary complications, as well as distinguishing AAAs from other etiologies. This narrative review provides a comprehensive exploration of the spectrum of imaging findings in atherosclerotic AAAs on CTA, underscoring the importance of structured reporting. Additionally, it examines therapeutic approaches and complications, and it differentiates AAAs from inflammatory, mycotic, and traumatic variants, serving as a primer for radiologists in AAA evaluation.

## 1. Introduction

**Figure 1:**
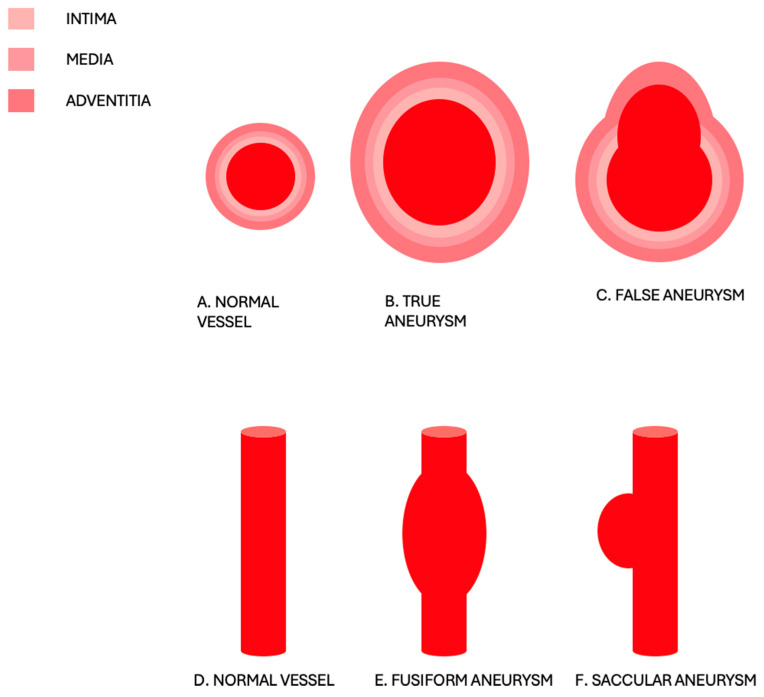
(**A**,**D**) Normal vessel. (**B**) True aneurysm in which all the layers of the wall are involved in the dilatation. (**C**) False aneurysm, also called pseudoaneurysm, in which the pathologic dilatation is encapsulated by the adventitia only. (**E**) Fusiform aneurysm, characterized by circumferential involvement of the artery, leading to a more spindle-shaped expansion around the vessel. (**F**) Saccular aneurysm, characterized by an asymmetrical, pouch-like bulge from only one side of the artery.

An abdominal aortic aneurysm (AAA) is defined as greater than 3 cm in maximum diameter or 50% larger than the normal proximal segment of the aorta. Aneurysms can be classified as either true or false based on the layers of the wall involved and as fusiform or saccular based on their morphology [1] (Figure 1).

Atherosclerotic AAAs are caused by the weakening of the aortic wall due to atherosclerosis, consisting in the accumulation of plaque within the arterial walls. Risk factors include male gender, white ethnicity, advanced age, a positive family history, tobacco use, and atherosclerosis [2,3].

The decreased prevalence and incidence rates of atherosclerotic AAAs over the past two decades have been attributed to the decrease in smoking and the enhancement of cardiovascular risk management through the pervasive use of statins and antiplatelets, as well as improved blood pressure control [4,5]. The most reliable evidence regarding the current prevalence of AAAs is derived from population screening studies. For instance, the Swedish Screening Programme reported a 1.7% prevalence in 65-year-old men between 2006 and 2009, with an additional 0.5% affected by an already known AAA [4], while the UK National Screening Programme 2009–2013 reported a prevalence of 1.3% [6]. In 2020 and 2021, both national screening programs reported a prevalence of less than 1%.

A ruptured abdominal aortic aneurysm (rAAA) represents a vascular surgical emergency, associated with a mortality rate of 80–90% [7].

Unruptured AAAs do not usually exhibit any noticeable symptoms. If symptoms or signs are present, they primarily manifest as pain or tenderness upon palpation of the AAA region. The symptoms may be caused by complications such as distal embolism or compression of the adjacent organs. When ruptured, the signs become evident and include pallor, hemodynamic collapse, abdominal and/or back pain, and abdominal distension [1].

AAAs are considered small when measuring between 30 and 55 mm and large when >55 mm [8].

**Figure 2:**
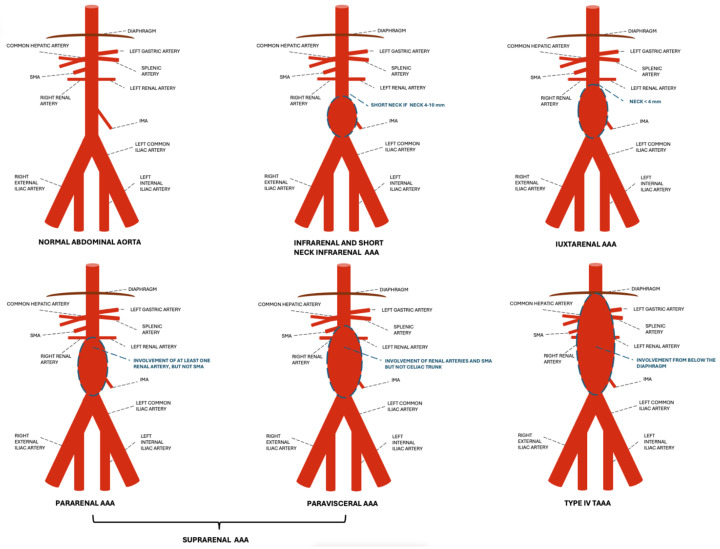
Schematic representation of a normal abdominal aorta and the different AAAs according to location. An infrarenal AAA has a neck > 10 mm in length, whereas the short-neck infrarenal AAA has a neck of 4−10 mm in length. A juxtarenal AAA has an aortic neck < 4 mm in length, without direct involvement of the renal arteries. A pararenal AAA involves at least one of the renal arteries but not the SMA. A paravisceral AAA involves the renal arteries and the SMA. Type IV AAAs involve the aorta from below the diaphragm. SMA: superior mesenteric artery, IMA: inferior mesenteric artery, AAA: abdominal aortic aneurysm.

Moreover, abdominal aortic aneurysms involving the renovisceral segment (without the involvement of the thoracic aorta) are collectively termed complex AAAs [8] (see Figure 2 for the classification of abdominal aneurysms).

## 2. Imaging Modalities

### 2.1. Ultrasonography

Ultrasonography (US) and duplex ultrasound (DUS) are the first-line diagnostic tools to detect and conduct surveillance on small AAAs [8,9]. Measurements must be performed in a plane perpendicular to the aortic longitudinal axis [10,11,12]. Different diameters can be measured but the anteroposterior is preferred over the other ones due to greater intra-observer coefficients of repeatability [13]. The use of a standardized US protocol, which includes electrocardiogram gating and offline reading with a small caliper, is recommended to reduce variability, as the measurement in diastole instead of systole can result in a 2 mm lower diameter due to the slight expansion of the aorta during systole from the increased blood pressure [14,15].

### 2.2. Computed Tomography

At present, computed tomography angiography (CTA) has emerged as the gold standard for pre-operative settings and the post-operative assessment of AAAs, given its ubiquity, and in cases of suspected rAAAs [1,8,16]. It is recommended that CTA be performed once the size threshold at which repair is considered has been reached, as determined by US, as CTA frequently overestimates the true diameter of AAAs, resulting in a higher percentage of referral to surgery [8,17,18].

The CTA scan should include the whole aorta, the supra-aortic vessels and the pelvic axis to identify concomitant aneurysms and assess significant stenoses along the commonly used transfemoral access points for endovascular aortic repair (EVAR), with a slice thickness of ≤1 mm to enable multiplanar reconstructions and facilitate detailed planning of endovascular procedures. While most aortic CTA protocols involve multiphase imaging, for pre-operative assessments (without clinical suspicion of contained rupture), thin-slice single arterial phase imaging (≤1 mm) is adequate for EVAR planning [9].

**Figure 3:**
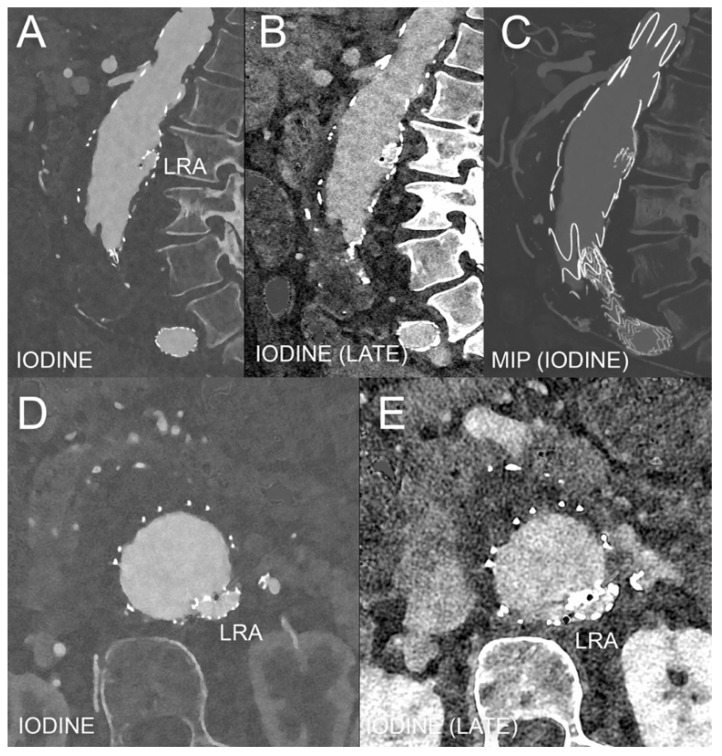
CT angiography of the abdomen using PCCT in the follow-up of an AAA treated with EVAR and with stent for the left renal artery to protect the left kidney. (**A**–**C**) show a sagittal reconstruction of the main axes of the aortic aneurysm, showing part of the lumen with the aortic endograft and part of the thrombus using a spectral iodine image in the arterial phase (**A**), a spectral iodine image in the late phase (**B**), and an MIP of the iodine image (**C**). The LRA in A indicates the region of the ostium of the stent inserted in the left renal artery to protect the left kidney. In (**D**,**E**), axial slices are shown using iodine images in the arterial phase (**A**) and late phase (**B**), again showing the diverse features of the lumen, the endograft, the thrombus, and the ostium of the LRA. The scan was performed on a commercial whole-body dual-source photon-counting CT scanner (Naeotom Alpha.Peak, Siemens Healthineers, Erlangen, Germany) with 0.2 mm slice thickness, 0.1 mm reconstruction increment, and 220 mm FOV; the scan was performed with spiral spectral acquisition and tube current modulation. Resolution matrix of 1024 × 1024 pixels on the source axial reconstructions with a kernel filtering of Bv48-60 (vascular kernel medium-sharp) and with maximum intensity of Quantum Iterative Reconstruction (QIR 4). The actual displayed resolution is 0.2 mm (200 microns). LRA: left renal artery; MIP: maximum intensity projection; FOV: field of view.

Dual-energy CT (DECT) allows the simultaneous or nearly simultaneous acquisition of CT images in low- and high-energy spectra, allowing materials to be distinguished based on their specific atomic numbers, unique k-edge properties, and varying linear attenuation coefficients [19]. DECT supports the generation of virtual non-contrast (VNC) images by subtracting iodine and iodine maps, which represent the distribution of iodine in tissues. VNC has been validated as an effective alternative to true non-contrast (TNC) imaging [20,21], offering the advantage of reduced radiation exposure, although vessel attenuation tends to be slightly higher with VNC compared to TNC [22,23]. Iodine maps, which can also be color-coded, are especially valuable in vascular imaging, such as for detecting endoleaks [24]. DECT also supports other reconstruction techniques, such as Virtual Monoenergetic Images (VMIs), simulating images acquired at a specific single-photon energy level, which reduce image noise and artifacts, including those caused by photon starvation and beam hardening, thereby improving image clarity and diagnostic accuracy [19]. Another notable algorithm is Virtual Noncalcium (VNCa) images, which removes calcified plaques while preserving intraluminal iodine-based contrast and surrounding soft tissue, making it especially useful for evaluating, for instance, carotid stenosis [25]. In Figure 3, see an example of DECT angiography, namely photon-counting CT (PCCT), in the follow-up of an AAA treated with EVAR.

### 2.3. Magnetic Resonance

Magnetic resonance angiography (MRA) is reserved for patients in whom the use of iodinated contrast agent or ionizing radiation should be avoided [26].

There are two broad categories within MRA to study vessels: black blood imaging, primarily used to visualize vessel walls and surrounding tissues rather than the blood flow itself, which appears dark on the images, and bright blood imaging, which studies blood flow within the vessels, highlighting the contrast between flowing blood and surrounding tissues, using either contrast-enhanced or non-contrast-enhanced techniques [27].

## 3. CTA Findings of Aneurysm Rupture

**Figure 4:**
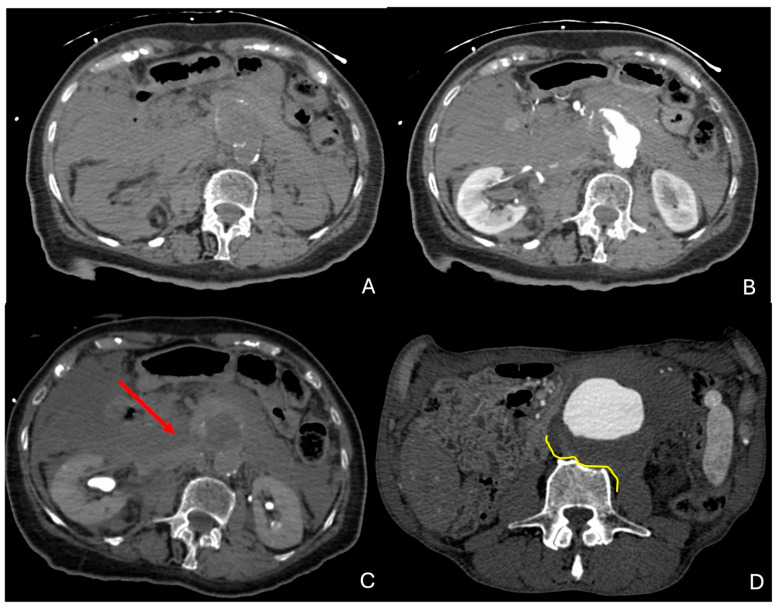
Signs of rupture of AAA. (**A**) Unenhanced CTA showing a large intra- and retroperitoneal hematoma with blood density in the context of a ruptured AAA of a patient admitted to the hospital for abdominal and back pain. (**B**,**C**) Arterial and delayed phase CTA showing contrast extravasation of the ruptured AAA in the same patient (red arrow). (**D**) Draped aorta (yellow line) with slight scalloping of the adjacent vertebra, in the context of a contained rupture in the retroperitoneum of another patient. CTA: computed tomography angiography; AAA: abdominal aortic aneurysm.

A retroperitoneal hematoma (Figure 4A–C) contiguous to an AAA and with possible extension into the pararenal and perirenal space is the hallmark of an rAAA. The extension into the intraperitoneal compartment can be apparent from the beginning or appear as a delayed finding. The collection of density of blood can be seen on unenhanced CT, whereas some contrast extravasation can frequently be appreciated on contrast-enhanced phases.

If the rupture is contained, “the draped aorta” sign (Figure 4D) can be observed, characterized by the posterior aortic wall losing its normal convex contour and instead appearing to “drape” over the vertebral column. This is indicative of the aortic wall being eroded or thinned out due to pressure, typically by a large aneurysm [28,29].

## 4. CTA Findings of Impending Aneurysm Rupture

### 4.1. Aneurysm Size

Aneurysm size is a strong determinant of rupture, according to the Laplace law T = PR, in which T is the circumferential wall tension, P the transmural pressure, and R the vessel radius [28].

In men, the risk of rupture is very low (0.3–0.8% per year) for AAAs with a diameter below 55 mm measured with US, which translates to a diameter on CTA between 55 and 62 mm depending on which measurement methodology is used. Consequently, it is unnecessary to reduce the diameter criterion for repair or to perform a CTA when ultrasound indicates a AAA diameter of less than 55 mm in males. Conversely, evidence from the UK screening program suggests increasing the diameter threshold for repair based on CTA to 60 mm [30,31].

In women, the risk of rupture for a small AAA is approximately four-fold greater than in males. The RESCAN meta-analysis indicates that the rupture rate for women with a 42 mm AAA is comparable to that of males with a 55 mm AAA, implying that a surgical intervention threshold of 45 mm may be suitable for women [32]. Nevertheless, the operational mortality rate is elevated in women compared to males for both endovascular and open repair procedures [33].

Currently, the guidelines of the European Society for Vascular Surgery (ESVS) [8] and Society for Vascular Surgery (SVS) [34] consider intervention for AAA > 55 mm in men and AAA > 50 in women, whereas those of the National Institute for Health and Care Excellence (NICE) [35] consider the same threshold of 55 mm for both women and men.

### 4.2. Expansion Rate

Another important predictor of rupture is the expansion rate. Three different patterns of growth have been described with abdominal aortic aneurysms: linear, staccato, and exponential [36].

Linear growth implies a steady, continuous increase in the size of the aneurysm over time, where the aneurysm diameter expands at a relatively constant rate. This pattern is considered more predictable, allowing regular monitoring to determine when repair should be performed.

Staccato growth involves sporadic and abrupt increases in aneurysm size, where periods of little or no growth are punctuated by sudden bursts of rapid expansion. This unpredictable nature increases concern, as rapid growth spurts can heighten the risk of rupture, even if the aneurysm had been stable for some time.

In exponential growth, the aneurysm grows at an increasing rate, with the expansion accelerating over time. Early growth might be slow, but as the aneurysm enlarges, its growth rate speeds up significantly, typically indicating a higher likelihood of rupture. This is considered the most dangerous growth pattern because the rapid expansion can be harder to control and manage without early surgical intervention.

A rate of expansion over 10 mm per year is associated with increased risk of rupture and warrants surgical repair, according to the guidelines by ESVS [8], SVS [34], and NICE [35].

### 4.3. Thrombus-to-Lumen Ratio

**Figure 5:**
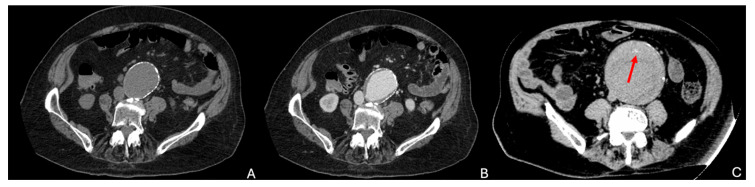
Signs of impending rupture. (**A**) Unenhanced CTA showing focal discontinuity of calcifications in a large infrarenal abdominal aneurysm (6.7 cm) in a patient with symptoms of abdominal pain. (**B**) Enhanced CTA showing the low thrombus-to-lumen ratio in the same patient. (**C**) Unenhanced CTA showing the hyper-attenuating crescent sign (red arrow). CTA: computed tomography angiography.

Unruptured AAAs generally contain more thrombus than ruptured aneurysms and the thrombus-to-lumen ratio decreases with increasing aneurysm size. Therefore, a thick circumferential thrombus is considered protective against rupture, whereas the enlargement of patent lumen, in turn related to partial lysis of the thrombus, is considered a predisposing factor of rupture [28,37] (Figure 5A,B).

### 4.4. Calcifications

The development of discontinuity in the aortic wall can also suggest either impending or ruptured aneurysms, especially when compared with a previous exam in which the calcification was circumferential [28,29] (Figure 5A,B).

### 4.5. Hyper-Attenuating Crescent Sign

This is identified as a crescent-shaped area of high attenuation (Figure 5C) within the thrombus or aneurysm wall, representing the presence of fresh intramural hemorrhage, and is highly indicative of impending rupture [28,37,38].

### 4.6. Primary Complications

**Figure 6:**
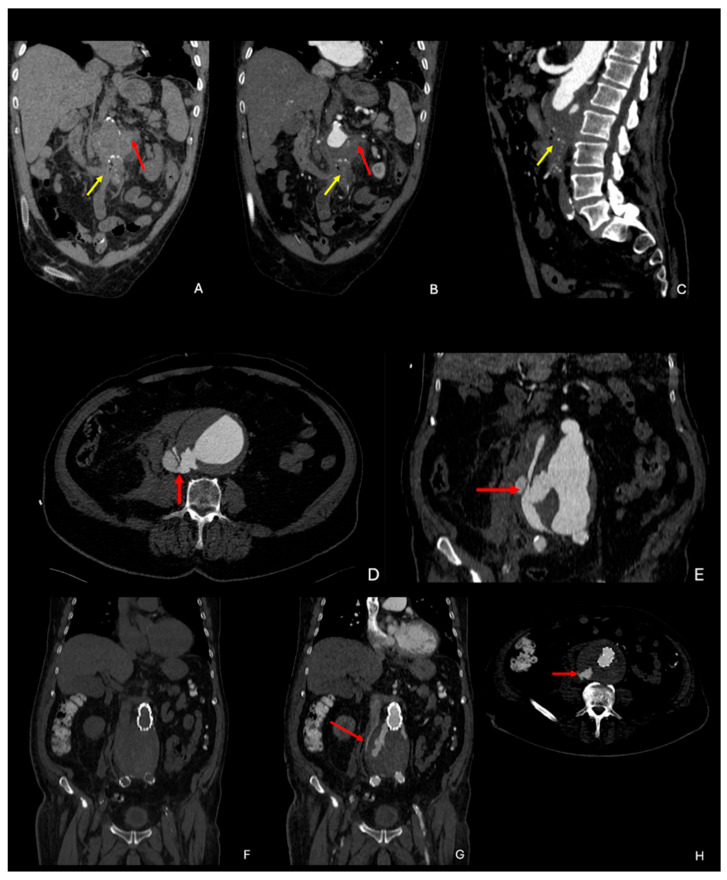
Primary complications of AAAs. (**A**) Coronal unenhanced CTA showing retroperitoneal hematoma (red arrow) with air bubbles due to primary aorto-enteric fistula (yellow arrow). (**B**) Coronal enhanced CTA showing retroperitoneal hematoma (red arrow) and air bubbles due to aorto-enteric fistula (yellow arrow). (**C**) Sagittal enhanced CTA showing retroperitoneal hematoma with air bubbles (yellow arrow) due to aorto-enteric fistula. (**D**,**E**) Axial and coronal enhanced CTA in arterial phase showing an aortocaval fistula (red arrow). (**F**–**H**) Coronal unenhanced CTA, and coronal and axial enhanced CTA in arterial phase, showing the same patient as in (**D**,**E**) after undergoing EVAR, with type 2 endoleak from the IMA and lumbar arteries, and the persistence, although smaller, of the aortocaval fistula (red arrow). CTA: computed tomography angiography; AAA: abdominal aortic aneurysm, IMA: inferior mesenteric artery.

Primary complications of AAAs include aorto-enteric fistula and aortocaval fistula. Aorto-enteric fistula (Figure 6A–C) is a pathologic connection, usually between the aorta and the third/fourth portion of the duodenum. CT imaging features include an abdominal aortic aneurysm, often with signs of rupture, and intraluminal and periaortic extraluminal gas. CTA may show contrast material extravasation from the aorta into the involved portion of the bowel, if a patent fistula is present. Aortocaval fistula (Figure 6D–H) is an exceedingly rare abnormal connection between the abdominal aorta and the inferior vena cava, with a reported incidence of <1%. It typically occurs without a significant hematoma, as the aortic rupture redirects blood flow directly into the caval vein. Unlike other aortic ruptures, aortocaval fistulas are generally not associated with substantial blood loss upon presentation. Instead, they commonly cause symptoms related to rapid arteriovenous shunting and right heart failure. On CTA, early opacification of the caval vein during the arterial phase may be observed [39].

The CTA findings of ruptured and unruptured AAAs with primary complications are summarized in Table 1.

## 5. Treatment

### 5.1. Procedure Types

When elective or emergency repair is selected, two options exist [8,34,35,40]: Open Surgical Repair (OSR) and Endovascular Aneurysm Repair (EVAR).

In OSR, polyester (Dacron) and expanded polytetrafluoroethylene (ePTFE) are commonly used materials for grafts. In OSR of AAAs, the proximal anastomosis is typically sutured near the renal arteries to minimize the risk of future aneurysm development [8,40,41].

In EVAR, the stent graft seals the aneurysm from the inside without removing or replacing the aneurysm wall, unlike in OSR. Devices use oversizing (typically between 10% and 25%) to ensure effective sealing and fixation, though this can vary depending on the specific stent graft used [8,40,41].

Aorto-uni-iliac grafts (AUI) [8] connect the aorta to a single iliac artery. Often, a femoral–femoral crossover bypass is performed simultaneously to maintain circulation to the contralateral leg. Aorto-bi-iliac grafts (ABI) [8] is a type of vascular graft that connects the aorta to both iliac arteries.

As for EVAR grafts, many techniques have emerged to maintain visceral branches that would be occluded with the placement of the endograft [8,40,42,43]:

- Chimney EVAR (CHEVAR): A conventional endograft is overlapped over coated stents, which are placed in the visceral branches or renal arteries, in order to maintain their patency while achieving an optimal seal zone.
- Fenestrated EVAR (FEVAR): These grafts have holes in the body, corresponding to the ostia of the visceral arteries and renal arteries involved in the aneurysm, in order to prevent them from becoming occluded. After placement of the fenestrated body, the covered stent is placed in the corresponding artery. These grafts are tailored to the patient’s anatomy, so they would not be available off-the-shelf in urgent cases.
- Branched EVAR (BEVAR): This is a main graft body to which secondary grafts are sewn to the main body.

**Figure 7:**
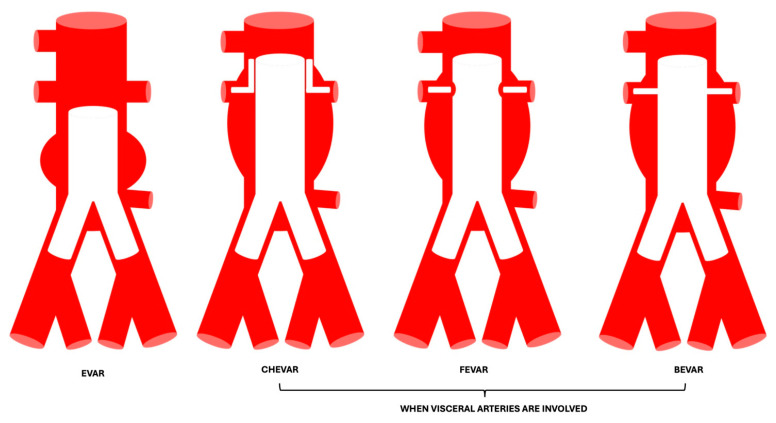
Schematic representation of different EVAR grafts. EVAR: endovascular aneurysm repair; CHEVAR: chimney EVAR; FEVAR: fenestrated EVAR; BEVAR: branched EVAR.

See Figure 7 for schematic representations of the different EVAR grafts.

### 5.2. OSR Versus EVAR: What the International Guidelines Say

According to the European guidelines [8], EVAR is preferred in most cases, but OSR is recommended for younger, fit patients (life expectancy > 10–15 years) or in cases of challenging anatomy (e.g., short/angulated landing zones). Complex cases may be treated with fenestrated/branched grafts.

According to the American guidelines [34], EVAR is preferred, with OSR for patients not meeting the anatomical criteria for EVAR.

Lastly, according to UK guidelines [35], EVAR benefits men > 70 and women of all ages, while OSR is preferred for men under 70.

## 6. Structured Reporting

When treating an AAA, whether by open surgery or endovascular repair, there are some key elements that need to be included in the diagnostic report.

### 6.1. Location

It is important to specify the location of the aneurysm, because the choice of the graft will depend on it. The location can be infrarenal or in cases of complex abdominal aortic aneurysms, short-neck infrarenal, juxtarenal, pararenal, paravisceral, and TAAAs [8,43,44].

### 6.2. Neck

The neck assessment is crucial because it impacts the suitability for EVAR. The neck should be sufficiently long (>10 mm) and have an adequate diameter (usually minimum diameter 16–19 mm and maximum 28–32 mm) to ensure proper graft fixation and avoid complications, such as endoleaks. The shape and presence of calcium and thrombus should be documented [8].

When measuring the neck of an abdominal aortic aneurysm (AAA), two critical angles are assessed: the alpha angle or suprarenal angle and the beta angle or infrarenal angle. The suprarenal angle is formed between the central axis of the suprarenal aorta and the proximal neck of the aneurysm. This angle should be in the range of 45–60° or not applicable, depending on some stent grafts. The infrarenal angle is created between the axis of the proximal neck and the axis of the lumen of the aneurysmal sac. This angle should be in the range of 45–90°, variable depending on neck length for some stent grafts. The neck is defined as hostile when length < 10 mm, diameter > 28 mm, alpha angle > 60°, in a conical configuration, or if there is significant mural thrombus of calcium [8,45].

### 6.3. Sac Assessment

It is important to specify the size of the aneurysmal sac, noting the presence of any mural thrombus and its distribution, particularly whether it is concentric. Additionally, the remaining lumen for blood flow should be measured. The length of the sac should be documented, including its starting point and where it terminates, especially if it involves the iliac bifurcation and visceral branches, along with the diameter of the aortic bifurcation, the length from lowest renal artery to the aortic bifurcation and the length from the lowest renal artery to the right common iliac bifurcation and left common iliac bifurcation, respectively. The aneurysmal sac can have various shapes, with fusiform being the most common, but it may also present as saccular [8,43,44].

### 6.4. Arterial Access

For EVAR procedures, a thorough evaluation of the femoral and iliac arteries is crucial to ensure that these arteries can accommodate the delivery and deployment of the endovascular graft. Potential obstructions, stenoses, or calcifications that might hinder access to the aortic aneurysm, along with artery size and tortuosity, must be evaluated to determine if they can safely serve as access routes for the procedure [8,43,44].

### 6.5. Other Points to Evaluate

Other important considerations to mention in the radiological report are (1) arterial stenosis of visceral arteries, such as the celiac trunk, SMA, or renal arteries, which can endanger organ viability; (2) anatomical variants, such as retroaortic renal veins; (3) accessory renal arteries; (4) co-existence of visceral aneurysms; (5) hypertrophic lumbar arteries which may lead to type 2 endoleaks; and (6) the co-existence of other processes, such as tumoral masses, complications from previous abdominal oncological therapies, or signs of previous surgeries, which may increase the morbidity risk attached to the procedure [8,43,44].

**Figure 8:**
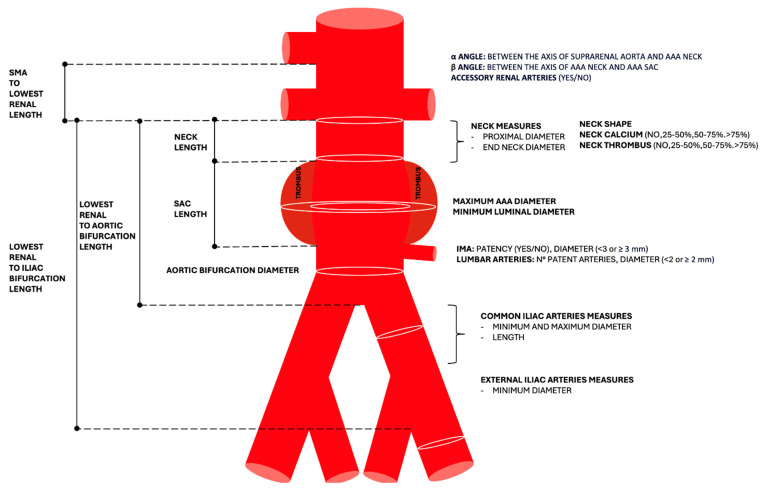
Schematic representation of values to cite in the structured radiology report. AAA: abdominal aortic aneurysm, IMA: inferior mesenteric artery.

See Figure 8 for a representative schematic report.

## 7. Secondary Complications

Secondary complications [46,47,48] can be divided into the following groups:

- Peri-operative complications: intra-abdominal hypertension (IAH) and intra-abdominal compartment syndrome (ACS), lower limb ischemia, and colonic ischemia;
- Late complications: while some complications are unique to one of the techniques (e.g., incisional hernias or para-anastomotic aneurysm formation after OSR or endoleak and stent migration after EVAR), others may occur irrespective of the technique used (e.g., graft infection, secondary aorto-enteric fistula, and graft occlusion). Patients treated by EVAR have a higher likelihood of experiencing aortic-related complications and requiring secondary interventions compared to those treated by OSR.

### 7.1. Graft Occlusion

**Figure 9:**
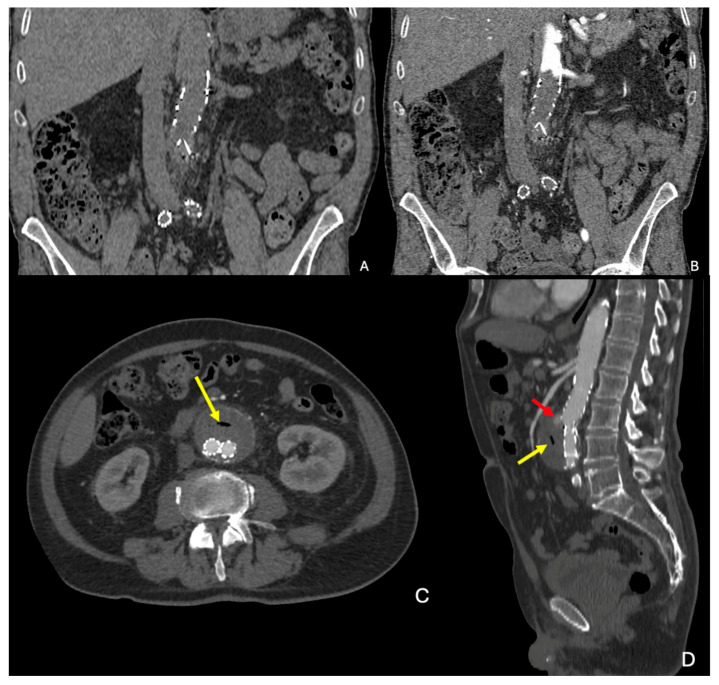
(**A**,**B**) Unenhanced and enhanced CTA showing a coronal view of a stent graft occlusion of a patient admitted to the emergency room for abdominal pain, who had undergone EVAR 4 years before. (**C**,**D**) Axial and sagittal enhanced CTA of another patient showing an infected EVAR graft with air bubbles (yellow arrow), mild fat stranding, and type 2 endoleak coming from the IMA (red arrow). IMA: inferior mesenteric artery.

This is a relatively frequent complication after OSR and EVAR (Figure 9A,B), accounting for roughly one-third of all secondary interventions. After OSR with a bifurcated prosthesis, limb occlusion occurs in 1–5% and after EVAR in 5.6% [8,49,50,51]. Radiologically, it appears as a non-enhancing concentric or eccentric thrombus along the internal wall of the endograft [52].

### 7.2. Graft Infection and Secondary Aorto-Enteric Fistulas

Prosthetic graft infection is a serious complication with a poor prognosis. It occurs in between 0.3% and 6% of cases after OSR and between 0.2 and 1% of cases after EVAR [8,53,54] (Figure 9C,D). Imaging features include fat stranding, fluid collection, contrast enhancement, and gas formation along the graft or pseudoaneurysm [55].

The reported frequency of secondary graft enteric fistula (GEF) is 0.3–4.3%, with a two-to-four-fold risk after OSR compared with EVAR [8,49].

### 7.3. Endoleak

Endoleaks represent a significant potential complication following EVAR, classified into five main types [8,40,56,57]:

- Type 1 endoleaks involve direct blood flow into the aneurysm sac due to insufficient seal at the stent graft’s proximal or distal attachment zones. It poses a high risk of rupture and occurs at three possible locations: type 1a, due to inadequate seal at the proximal end; type 1b, due to distal end inadequacy; and type 1c, which occurs at an iliac occluder following aorto-uni-iliac (AUI) repair with femorofemoral crossover graft.
- Type 2 endoleaks: the most common form, arising from collateral vessel backflow, especially from lumbar arteries or the inferior mesenteric artery (IMA). Risk factors include the presence of patent aortic side branches, IMA diameter over 3 mm, patent lumbar arteries (more than three, or over 2 mm in diameter), and anticoagulant use. Embolization of these vessels or non-selective sac embolization can reduce the occurrence of type 2 endoleaks.
- Type 3 endoleaks: these result from stent graft component separation or a tear in the graft fabric. Causes include stent graft migration, inadequate overlap between components (Type 3a), or material fatigue (Type 3b). Component separation can precede the development of an endoleak.
- Type 4 endoleaks: rarely observed with modern graft materials, type 4 endoleaks involve blood leakage through the stent graft due to graft porosity. These leaks usually appear shortly post-operatively and are generally benign and transient.

Sometimes sac growth is noticed without any detectable endoleak, suggesting the presence of non-visible pressurization, and these cases are termed type 5 endoleaks. This remains a subject of ongoing investigation due to its unclear mechanism and lack of visibility on imaging.

**Figure 10:**
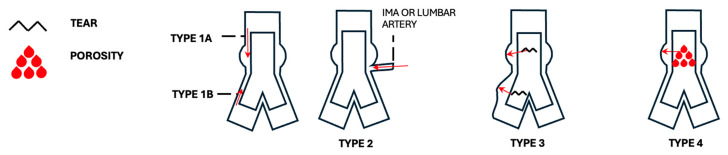
Endoleaks. Notes to the figure: type 1C is not depicted, nor type 5.

**Figure 11:**
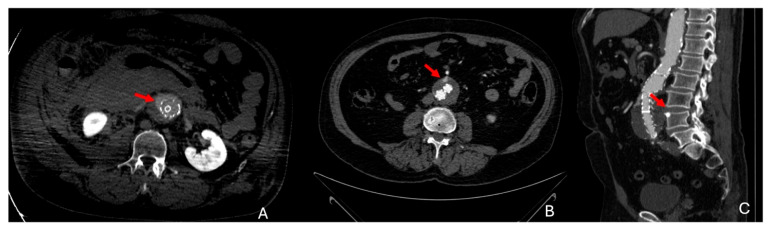
(**A**) Axial enhanced CTA in arterial phase, showing type 1A endoleak in a patient who had recently undergone EVAR for rAAA (red arrow). (**B**,**C**) Axial and sagittal enhanced CTA in arterial phase type 2 endoleak from the IMA (red arrow). EVAR: endovascular aneurysm repair, rAAA: ruptured abdominal aortic aneurysm, IMA: inferior mesenteric artery.

See Figure 10 for an illustration of the endoleak subtypes, Figure 11A for an example of type 1 endoleaks, and Figure 11B,C for type 2 endoleaks.

### 7.4. Stent Migration

The incidence of stent migration after EVAR is reported to be between 1% and 10% of patients. Factors influencing migration include the design of the stent graft and the anatomical characteristics of the aorta. Migration can lead to complications such as endoleaks or compromised blood flow, necessitating further intervention [8,52]. Device migration is defined as device movement of more than 10 mm on the centerline or movement of more than 15 mm on either the anterior or posterior aortic margin [58].

### 7.5. Para-Anastomotic Aneurysm

**Figure 12:**
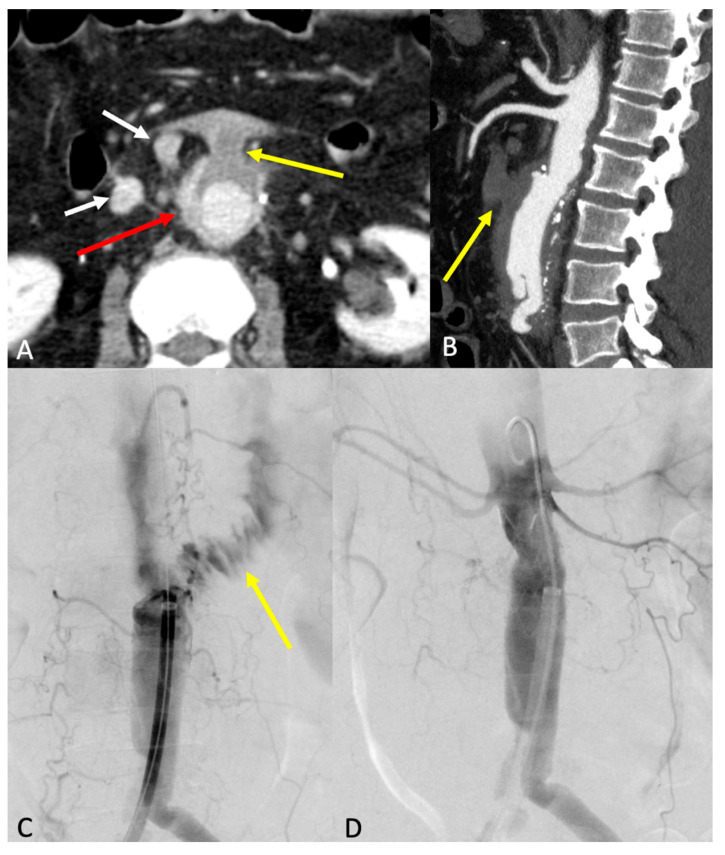
Outcomes of aorto-iliac graft surgery for abdominal aortic aneurysm in CTA (**A**,**B**) and angiography (**C**,**D**). The images show an infected proximal anastomotic pseudoaneurysm (red arrow in (**A**)) characterized by a partially thrombosed aorto-duodenal fistula (yellow arrow in (**A**,**B**)) and periaortic lymph nodes (white arrows in (**A**)). The angiographic exam shows the passage of the contrast medium into the duodenum (yellow arrow in (**C**)) and the subsequent positioning of the endoprosthesis to seal the fistula (**D**).

This occurs either as a true aneurysm forming near the anastomosis or as a false aneurysm resulting from anastomotic disruption, often attributed to an underlying graft infection (Figure 12A–D) [8].

## 8. Differential Diagnosis

Whereas atherosclerotic abdominal aortic aneurysms are more frequent, atherosclerosis is not the sole cause of an abdominal aortic aneurysm.

### 8.1. Infected Aneurysm

Mycotic aneurysms, also called infective native aortic aneurysms, are typically pseudoaneurysms rather than true aneurysms, arising secondary to infection rather than degenerative vascular disease [59]. In Western regions, they represent 0.5% to 2.6% of aortic aneurysms and often affect younger individuals. Mycotic aneurysms tend to occur in immunocompromised patients or those with systemic infections (e.g., osteomyelitis, tuberculosis, or urinary infections) or conditions like diabetes, renal disease, or HIV [60]. The most implicated pathogens in mycotic aneurysms are Staphylococcus aureus and coagulase-negative Staphylococcus species. These aneurysms frequently appear in the thoracic or suprarenal aorta and are associated with high rupture rates, with over 44% presenting with rupture at diagnosis [61].

Diagnosing mycotic AAAs requires combining clinical presentation, microbiology, and radiological findings. A characteristic appearance on CTA includes a saccular or lobular shape, periaortic inflammation, and potential signs of infection like periaortic gas or abscesses. Faster growth rates in saccular aneurysms should raise suspicion for infection, particularly if CTA reveals rapid expansion. In challenging cases, molecular imaging, such as F-fluoro-2-deoxyglucose (^18^FDG) positron emission tomography (PET) or white blood cell scans, can help identify active infections [62,63,64].

Timely diagnosis, prompt initiation of intravenous antibiotics, and early surgical intervention are crucial for managing mycotic aneurysms, regardless of size. These aneurysms exhibit unpredictable progression, and delaying surgery increases the risk of rupture and mortality [8,65,66].

### 8.2. Inflammatory Aneurysm

Inflammatory AAAs constitute a distinct category of abdominal aortic aneurysms, accounting for approximately 5–10% of cases. They typically occur in younger patients compared to degenerative AAAs and are more common in males, particularly heavy smokers with pre-existing conditions such as hypertension, coronary artery disease, or peripheral artery disease [67]. Unlike typical degenerative AAAs, inflammatory AAAs are associated with chronic periaortitis, which is characterized by marked thickening of the aneurysm wall, perianeurysmal fibrosis, and dense adhesions to nearby structures [68].

The etiology of inflammatory AAAs remains unclear, but evidence points to an autoimmune response, likely triggered by a reaction to components of atherosclerotic plaques or as part of a systemic inflammatory disease [69]. These aneurysms are sometimes associated with elevated immunoglobulin G4 (IgG4) levels, which may correlate with other systemic IgG4-related diseases, and large-vessel vasculitis. Patients often present with symptoms more frequently than degenerative AAAs, including abdominal or back pain, weight loss, and elevated inflammatory markers [70].

**Figure 13:**
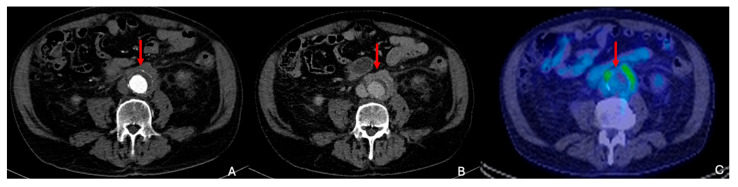
Inflammatory AAAs. (**A**) Arterial phase CTA imaging demonstrates an infrarenal AAA measuring 3.5 cm, accompanied by visible periaortic tissue (red arrow). (**B**) The periaortic tissue (red arrow) exhibits enhancement in the delayed phase of CTA, commonly referred to as the “mantle sign”. (**C**) Fused PET-CT images reveal significant ^18^F-FDG uptake within the periaortic tissue (red arrow). Subsequent evaluation identified the inflammatory aneurysm as being associated with IgG4-related disease.

CTA is the primary diagnostic tool for inflammatory AAAs, often revealing the characteristic “mantle sign”, or thickened periaortic tissue encasing the aneurysm, and ^18^FDG PET can identify inflammatory tissue [64,71] (Figure 13).

Although these aneurysms have a low rupture risk (less than 5%), close monitoring is essential, and surgery may be necessary when aneurysm growth or symptoms become unmanageable with medical treatment alone [8,67]. Because of the complex presentation, diagnosis, and management of inflammatory AAAs, a multidisciplinary approach is recommended [72].

### 8.3. Genetic Syndromes

In younger individuals with AAAs or those with a family history or signs of monogenic syndromes, a targeted diagnostic strategy is essential. Unlike typical AAAs associated with cardiovascular risk factors, these cases may stem from genetic or connective tissue disorders that weaken blood vessels and promote aneurysm formation. More than 30 inherited conditions, including Marfan syndrome, vascular Ehlers–Danlos syndrome (vEDS), Loeys–Dietz syndrome, arterial tortuosity syndrome, and aneurysm–osteoarthritis syndrome, have been linked to arterial aneurysms, though they more commonly affect the thoracic aorta than the abdominal aorta. A multidisciplinary aortic team typically evaluates management strategies for these patients, with imaging modalities such as CTA, MRA, and DUS playing a key role in monitoring and assessment [8,73,74,75].

### 8.4. Traumatic Aneurysms

These are rare entities, usually pseudoaneurysms, caused for instance by blunt abdominal aortic injury (1:20 when compared with traumatic thoracic pseudoaneurysms) [76].

See Table 2 for a summary of the different etiologies of abdominal aortic aneurysms.

## 9. Future Directions

Artificial Intelligence (AI) refers to the capability of computational systems to perform tasks typically associated with intelligent beings. Within the realm of AI, machine learning (ML) stands out as a technique that allows for the identification of patterns and decision-making from large datasets, without relying on explicit instructions or predefined assumptions. Machine learning is generally classified into two primary categories: unsupervised learning and supervised learning [77].

AI applications have been explored across various medical fields, including imaging and biological analysis, offering potential advancements in patient diagnosis, prognosis, and treatment [78].

In the context of AAAs, AI proves to be a valuable tool for interpreting and analyzing imaging, facilitating automatic quantitative measurements [79,80] and morphologic and hemodynamic characterization [81,82]. This capability supports surgeons in pre-operative planning and in developing computational models to predict AAA progression [83], risk of rupture [84], and post-operative outcomes [85,86].

For instance, Lee et al. [83] employed nonlinear kernel Support Vector Regression (SVR) to predict aneurysm growth, with the model successfully estimating the individual’s AAA diameter to within a 2 mm margin of error in 85% and 71% of patients at 12 and 24 months, respectively.

Similarly, Karthikesalingam et al. [85] developed an artificial neural network (ANN) model based on aortic morphological features to assess the risk of complications from EVAR. In a cohort of 761 patients with a mean follow-up of 38 ± 20 months, the ANN model was able to predict endograft complications and mortality, accurately distinguishing between low- and high-risk patients.

## 10. Conclusions

The early diagnosis and treatment of AAAs, whether atherosclerotic, inflammatory, or mycotic, are crucial for reducing the risk of severe complications, particularly rupture, which has a high mortality rate. Effective diagnosis, often involving ultrasound and/or CT, enables timely intervention tailored to the aneurysm subtype, size, and patient fitness, whether through OSR or EVAR.

CT imaging is essential not only for diagnosing AAA but also for post-operative management and monitoring after AAA repair. Following surgery, CTA scans help detect potential complications such as endoleaks, stent migration, graft occlusion, graft infection and aorto-enteric fistulas, all of which may reveal catastrophic if not promptly found and addressed.

Additionally, AI can streamline the interpretation of CTA scans and assist clinicians, providing not only automatic quantitative measurements but also contributing to predictive models for progression, pre-operative and post-operative complications.

## Figures and Tables

**Table 1:** CTA findings of ruptures and impending ruptures of AAAs and their primary complications.

	CTA Findings
Rupture	- Retroperitoneal hematoma, with possible contrast extravasation into the hematoma - “Draped aorta” sign if rupture is contained
Impending rupture	- Discontinuity of calcification - Low thrombus-to-lumen ratio - Crescent hyper-attenuation sign - Large AAA - AAA with exponential or staccato growth when compared with previous CTA
Aorto-enteric fistula	- Abdominal aortic aneurysm, often with signs of rupture - Intraluminal and periaortic extraluminal gas - Contrast material extravasation from the aorta into the involved portion of the bowel, if a patent fistula is present
Aortocaval fistula	Early opacification of vena cava

**Table 2:** Table summarizing key points of the different subtypes of AAAs.

AAA Subtype	Demographics	Pathogenesis	Key Features	Imaging Findings
Atherosclerotic	Typically affects older adults Male predominance	Caused by degeneration of the aortic wall, related to atherosclerosis	- Asymptomatic or symptomatic (with or without rupture) - Risk of rupture	- Dilated aorta - Mural thrombus - Wall calcifications - Crescent sign if hemorrhage in wall or thrombus - Discontinuity of the wall and contrast extravasation if ruptured - Draped aorta sign if rupture is contained or imminent rupture
Infected	In the context of systemic infection, more common in immunocompromised patients	Secondary to bacterial infection	- Fever - Abdominal pain - Elevated inflammatory markers - Risk of rapid expansion and rupture	Saccular aneurysm with periaortic gas, inflammatory signs ^18^FDG PET: Increased uptake in infected areas
Inflammatory	Affects younger males (50–65 years), often heavy smokers	Unknown etiology; suspected autoimmune response	- Abdominal, back, or flank pain, weight loss - Elevated inflammatory markers	- Periaortic soft tissue, retroperitoneal fibrosis - ^18^FDG PET: Identifies inflammatory activity
Genetic	- Younger patients < 60 years with connective tissue disorders - Positive family history	Caused by genetic mutations	- Higher rupture risk at smaller diameters - Features like joint hypermobility, skin elasticity	- CTA: Tortuous vessels, aneurysm with thin walls - MRA: Preferred in patients needing lifetime imaging to avoid radiation exposure
Traumatic	- Can occur at any age following blunt trauma or injury - No sex preference	Caused by direct vessel wall injury or pseudoaneurysm formation	- Acute abdominal/back pain following trauma - Potential for sudden rupture	- Saccular aneurysm with surrounding hematoma, irregular wall

^18^FDG PET: F-fluoro-2-deoxyglucose positron emission tomography; CTA: computed tomography angiography, MRA: magnetic resonance angiography.

## Data Availability

No new data were created or analyzed in this study. Data sharing is not applicable to this article.

## Abbreviations

The following abbreviations are used in this manuscript:

AAA	Abdominal aortic aneurysm
US	Ultrasound
DUS	Duplex ultrasound
CTA	Computed tomography angiography
AI	Artificial intelligence
